# 18F-Fluorodeoxyglucose Positron Emission Tomography in Elderly Patients with an Elevated Erythrocyte Sedimentation Rate of Unknown Origin

**DOI:** 10.1371/journal.pone.0058917

**Published:** 2013-03-19

**Authors:** Karel-Jan D. F. Lensen, Alexandre E. Voskuyl, Conny J. van der Laken, Emile F. I. Comans, Dirkjan van Schaardenburg, Alex B. Arntzenius, Ton Zwijnenburg, Frank Stam, Michelle Gompelman, Friso M. v.d. Zant, Anneke Q. A. van Paassen, Bert J. Voerman, Frits Smit, Sander Anten, Carl E. Siegert, Arjen Binnerts, Yvo M. Smulders

**Affiliations:** 1 Department of Internal Medicine and the Institute for Cardiovascular Research (ICaR-VU), VU University Medical Center Amsterdam, Amsterdam, The Netherlands; 2 Department of Rheumatology, VU University Medical Center, Amsterdam, The Netherlands; 3 Department of Nuclear Medicine, VU University Medical Center, Amsterdam, The Netherlands; 4 Department of Rheumatology, Jan van Breemen Institute/Reade, Amsterdam, The Netherlands; 5 Department of Internal Medicine, Spaarne Hospital, Hoofddorp, The Netherlands; 6 Department of Nuclear Medicine, Spaarne Hospital, Hoofddorp, The Netherlands; 7 Department of Internal Medicine, Medical Center Alkmaar, Alkmaar, The Netherlands; 8 Department of Nuclear Medicine, Medical Center Alkmaar, Alkmaar, The Netherlands; 9 Department of Internal Medicine, Kennemer Gasthuis, Haarlem, The Netherlands; 10 Department of Internal Medicine, Amstelland Hospital, Amstelveen, The Netherlands; 11 Department of Internal Medicine, Rijnland Hospital, Leiderdorp, The Netherlands; 12 Department of Nuclear Medicine, Rijnland Hospital, Leiderdorp, The Netherlands; 13 Department of Internal Medicine, Sint Lucas Andreas Hospital, Amsterdam, The Netherlands; 14 Department of Internal Medicine, Zaans Medical Center, Zaandam, The Netherlands; University of Manchester, United Kingdom

## Abstract

Patients with an elevated erythrocyte sedimentation rate (ESR) and non-specific symptoms often pose a diagnostic dilemma. PET/CT visualises infection, inflammation and malignancy, all of which may cause elevated ESR. The objective of this study was to determine the contribution of 18F-fluorodeoxglucose positron emission tomography (PET/CT) in the diagnostic work-up of referred patients with an elevated ESR, in whom initial routine evaluation did not reveal a diagnosis. We conducted a combined retrospective (A) and prospective (B) study in elderly (>50 years) patients with a significantly elevated ESR of≥50 mm/h and non-specific complaints. In study A, 30 patients were included. Malignancy (8 patients), auto-inflammatory disease (8 patients, including 5 with large-vessel vasculitis) and infection (3 patients) were suggested by PET/CT. Two scans showed non-specific abnormalities and 9 scans were normal. Of the 21 abnormal PET/CT results, 12 diagnoses were independently confirmed and two alternative diagnosis were made. Two diagnoses were established in patients with a normal scan. In study B, 58 patients in whom a prior protocolised work-up was non-diagnostic, were included. Of these, 25 PET/CT-scans showed suspected auto-inflammatory disease, particularly large-vessel vasculitis (14 cases). Infection and malignancy was suspected in 5 and 3 cases, respectively. Seven scans demonstrated non-specific abnormalities, 20 were normal. Of the 40 abnormal PET/CT results, 22 diagnoses were confirmed, 3 alternative diagnoses were established. Only one diagnosis was established in the 20 patients with a normal scan. In both studies, the final diagnosis was based on histology, clinical follow-up, response to therapy or additional imaging. In conclusion, PET/CT may be of potential value in the diagnostic work-up of patients with elevated ESR if routine evaluation reveals no diagnosis. In particular, large-vessel vasculitis appears to be a common finding. A normal PET/CT scan in these patients suggests that it is safe to follow a wait-and-see policy.

## Introduction

Patients with elevated inflammatory parameters pose a common diagnostic dilemma for many clinicians. The erythrocyte sedimentation rate (ESR) is often determined in patients presenting with non-specific signs and symptoms like malaise, fatigue, weight loss or anorexia.[1], [2] A persistently marked elevation of ESR suggests chronic inflammatory or malignant diseases.[3] However, initial tests, such as screening for infections, paraproteinemia, and cancer (classically renal cell carcinoma) by chest X-ray and abdominal ultrasound, often turn out negative. In this situation, there is no consensus on how to proceed.

18F-fluorodeoxyglucose positron emission tomography combined with low-dose computed tomography (PET/CT) is a hybrid imaging technique displaying high metabolic turnover of both physiologic (myocardial and cerebral glucose uptake) and non-physiologic origin.[4] PET/CT may have diagnostic value in patients with non-specific complaints and an elevated ESR by showing abnormal 18F-FDG uptake suggestive of infection, malignancy or auto-inflammatory disease, such as sarcoidosis or large-vessel vasculitis. In fever of unknown origin, a related condition that also raises suspicion of inflammatory disease, PET/CT contributes to establishing a diagnosis in 36–69% of cases.[5]–[9] Causes of fever of unknown origin and elevated ESR only partly overlap.[10] A study systematically evaluating PET/CT as a diagnostic tool for elevated ESR in patients without fever has never been performed.

Therefore, the objective of our hospital-based study was to determine the contribution of PET/CT in the diagnostic work-up of patients referred with a markedly elevated ESR, in whom the initial routine evaluation did not reveal a diagnosis.

## Methods

### Study design

The results of two studies are reported: a retrospective and a prospective analysis of the diagnostic yield of PET/CT in patients older than 50 years with an elevated ESR≥50 mm/h. An age cut-off of 50 years was chosen based on the age-relatedness of several conditions that may show up on PET/CT, such as cancer and giant cell arteritis/large-vessel vasculitis. Also, the diagnostic dilemma of elevated ESR is more common in elderly subjects.[1] The studies were performed in a tertiary referral center and seven community medical centers.

### Ethics statement

The Medical Ethics Committee of the VU University Medical Center approved the protocol. The institutional review board waived the need for written informed consent from the participants as all procedures were performed as part of clinical care.

### Patient population

#### Retrospective study

Patients were selected by reviewing all consecutive PET/CT application forms that were received at the departments of nuclear medicine of participating community medical centers during a six-month period. One investigator (KL) checked all application forms for scan-indication. Patients were considered to be potentially eligible if either an elevated ESR or ‘elevated inflammatory markers’ was mentioned as indication for PET/CT. Subsequently, hospital records of these patients were used to retrieve clinical data. Patients older than 50 years were included if the ESR was higher than 50 mm/h (Westergren method) on at least one occasion before PET/CT was performed. Patients were excluded if fever was present or if their medical history or physical examination revealed potentially diagnostic clues (PDC’s), e.g. headache suggesting temporal arteritis. Clinical signs and symptoms, present before the PET/CT scan was performed, were evaluated as PDC’s during a consensus meeting, during which the results of the PET/CT scan were not known.

#### Prospective study

In the prospective study, consecutive eligible patients (out- or inpatients) were included if they were older than 50 years, and had an ESR≥50 mm/h documented on at least two separate occasions, preferably at least four weeks apart. Shorter measurement intervals were accepted at the discretion of the treating physician, e.g. when clinical signs or symptoms were present for more than 4 weeks but ESR had not been determined when signs or symptoms initially developed. Finally, a non-diagnostic chest X-ray, abdominal ultrasound and absence of or minor (i.e. non-contributing) paraproteinemia had to be present prior to the PET/CT-scan. Any alternative examination (e.g. CT-scan) was performed at the discretion of the treating physician and had to be non-diagnostic. Patients were not included if their history or physical examination revealed PDC’s or if fever was present. Patients were recruited from departments of internal medicine and rheumatology.

In both studies patients were excluded when using immunosuppressive drugs (e.g. corticosteroids) at the time of PET/CT-scanning, as such therapy has been reported to decrease sensitivity of 18F-FDG uptake.[11]

### 18F-FDG PET/CT-protocol

A protocol for 18F-FDG PET/low-dose CT scanning has been developed specifically for multi-center clinical trials in the Netherlands.[12] In the prospective study all patients were scanned according to this protocol. In short, the protocol provides guidelines on patient preparation, dose and method of administration of 18F-FDG, post-injection scan time and scan characteristics (i.e. amount of min/bed depending on bed overlap and mode of acquisition (2D or 3D) and image reconstruction) for all available PET/CT-scanners. In our patients, after fasting for at least four hours, whole-body (from head to knees) PET-scans were acquired 60 (±5) minutes after intravenous injection of 1,25−5 MBq/kg 18F-FDG. A low-dose CT-scan was acquired prior to the PET-emission scan for anatomic localization and attenuation correction. In the retrospective study all patients were scanned before initiation of the study. Therefore, PET/CT-scans were performed according to local protocols in this study. These protocols are well suited for the (non-quantitative) visual assessment of 18-FDG PET/CT images. On the contrary, quantitative assessment of images (using standardized uptake values or SUV’s) are affected by variations in protocol. Therefore, visual assessment was the method of choice in this study.

Two different types of scanners were used in both studies (Gemini TOF-16; Philips, The Netherlands and Biograph 2; Siemens, Germany).

### Image analysis

All scans were performed as part of clinical assessment and the study was designed to resemble daily clinical practice as much as possible. Accordingly, the assessment of non-physiological 18F-FDG uptake was done by the attending nuclear medicine physicians, and observers were not blinded for clinical data. The interpretation of 18F-FDG uptake was based on visual (qualitative) characteristics.

### Follow-up

For both the retrospective and the prospective study follow-up data were acquired to determine whether additional investigation(s) (e.g. histopathology) or clinical management (e.g. clinical response to therapy) could confirm the diagnosis that was suspected based on PET/CT results. Likewise, follow-up data were used to assess whether a diagnosis had been established in patients with a normal PET/CT-scan, or whether an alternative diagnosis was obtained in patients with an abnormal PET/CT-scan. Hospital records were reviewed for this purpose. The general practitioner was contacted to obtain missing information if the hospital records contained insufficient data. Patients were required to have at least 3 months of follow up information to be included in this analysis. If follow-up data at 3 months after PET/CT was insufficient, patients were considered lost-to follow-up.

## Results

**Figure 1:**
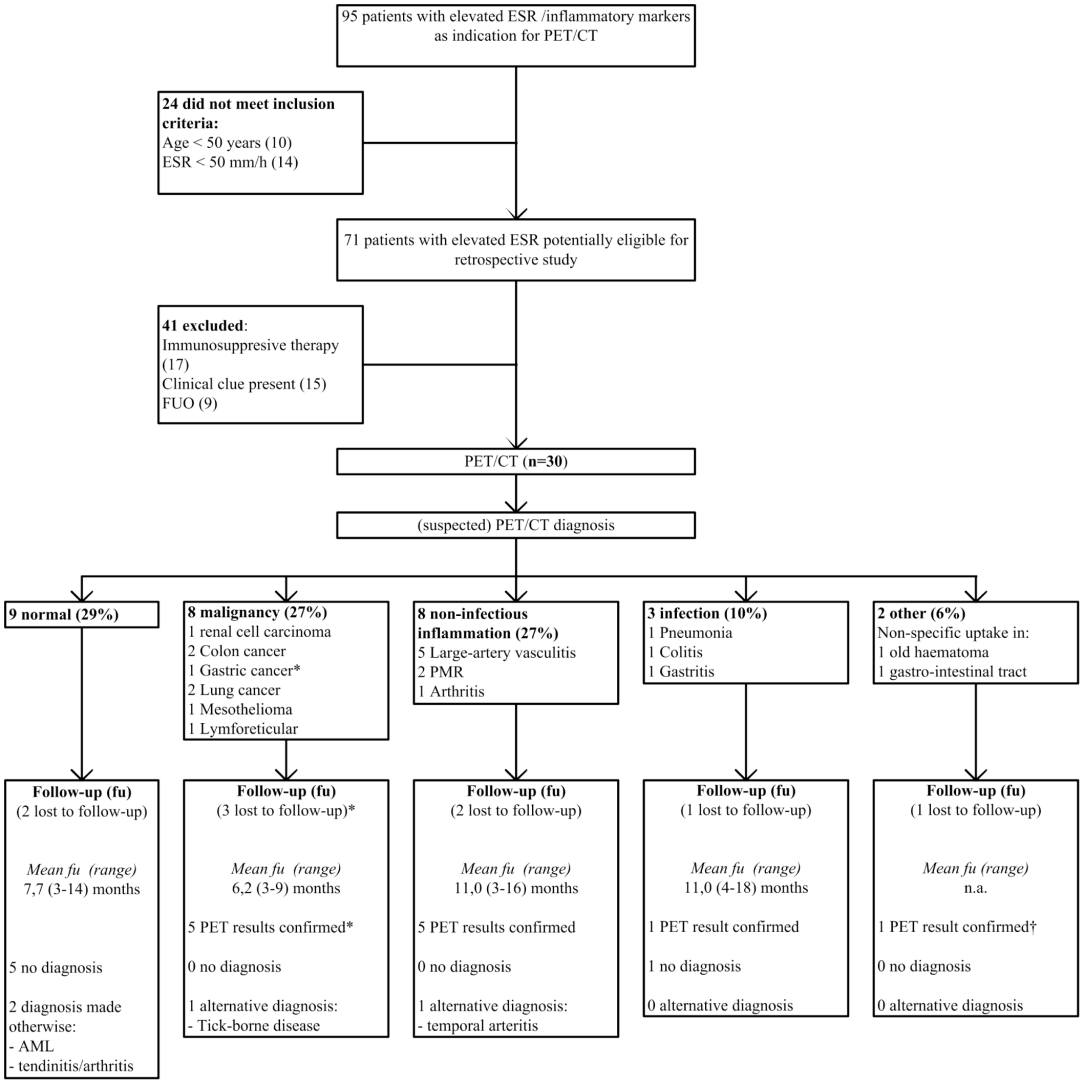
Flowchart retrospective study (ESR = Erythrocyte Sedimentation Rate; FUO = Fever of Unknown Origin, PMR  =  Polymyalgia rheumatica, AML = acute myeloid leukemia, n.a. =  not applicable). *This patient died 1 month after PET/CT, gastric cancer was confirmed pathologically. † Polyarteritis nodosa (diagnosed with angiography).

**Figure 2:**
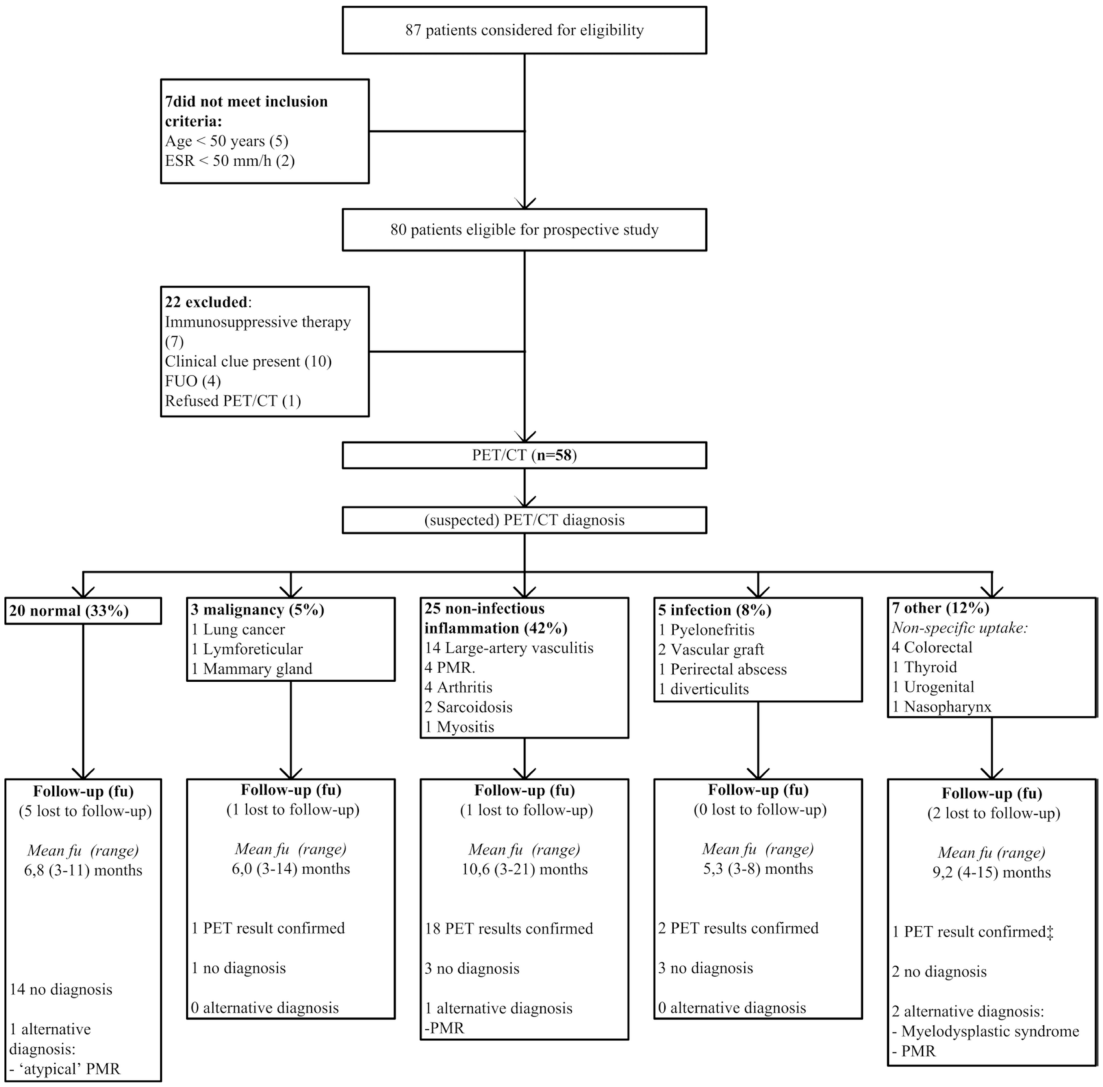
Flowchart prospective study (ESR = Erythrocyte Sedimentation Rate; FUO = Fever of Unknown Origin, PMR  =  Polymyalgia rheumatica). ‡chronic tonsillitis (diagnosed by ear-nose-throat specialist).

Baseline demographics and clinical characteristics of patients in both studies are displayed in Table 1. Mean age and ESR were similar in both studies. A larger proportion of women was included in the prospective study. Also, the prospective study comprised more patients complaining from night sweats. Finally, non-specific complaints of the locomotor tract were encountered more frequently in the prospective group, which is probably a reflection of a higher number of patients recruited at Rheumatology departments. Flowcharts for inclusion, PET/CT- and follow-up results for both studies are shown in Figures 1 and 2.

**Table 1:** Baseline demographics and clinical characteristics.

	Retrospective study (n = 30)	Prospective study (n = 58)
ESR (mm/h)	87 (24)	85 (22)
Age (years)	70 (11)	72 (11)
Sex (male/female)	14/16	22/36
*General symptoms*		
Weight loss	37%	38%
Anorexia	23%	26%
Night sweats	3%	12%
*Local symptoms*	29%	45%
Locomotor	13%	29%
Myalgia	0%	8.5%
Arthralgia	0%	12%
Non-specific pain	13%	8.5%
Gastro-intestinal	7%	7%
Respiratory	3%	3%
Non-specific headache	3%	3%
Other	3%	3%

Continuous data are presented as mean (SD). ESR = Erythrocyte Sedimentation Rate.

### Retrospective study

As shown in Figure 1, 95 patients were considered for inclusion, of whom 30 were included. Examinations performed prior to PET/CT in these patients are reported in Table 2. A thoracic CT and an abdominal CT were each performed in 1 patient and revealed no diagnostic clues. Overall, 21 of 30 (71%) PET/CT-scans were judged as abnormal. PET/CT mainly raised suspicion of malignancy (8/30, 27%) and auto-inflammatory disease (8/30, 27%). Infections were detected in 3/30 (10%) of PET/CT scans, whereas non-specific abnormal 18F-FDG uptake was seen in 2/30 (6%) PET/CT-scans.

**Table 2:** Examinations performed prior to PET/CT scan in patients included in retrospective study.

	Retrospective study (n = 30)	Prospective study (n = 58)
Chest X-ray	68%	98%
Abdominal ultrasound	64%	97%
Protein electrophoresis	54%	98%
Abdominal CT	3%	10%
Thoracic CT	3%	10%

PET/CT results suggested 14 different individual diagnoses. Large-vessel vasculitis was suspected most frequently (5 of 21 abnormal scans).

Among 21 patients with sufficient follow-up data, diagnoses suspected based on PET/CT results were confirmed in 14 of 16 cases (88%) during follow-up (5 patients had a normal scan result). Furthermore, an alternative diagnosis was made in 2 of 16 patients (13%) with an abnormal PET/CT result. One patient was eventually diagnosed with temporal arteritis, (diagnosed clinically as the patient refused a temporal artery biopsy, the scan result suggested polymyalgia rheumatic (PMR)), whereas the other was diagnosed with tick-borne disease (the scan result was suspected lung cancer). In the 9 patients with a normal PET/CT, a diagnosis was obtained during follow-up in 2. Tendinits/arthritis was diagnosed in one, acute myeloid leukaemia (based on bone marrow examination) in the other. Polyarteritis nodosa was diagnosed (angiographically) in the patient showing non-specific uptake in the gastro-intestinal tract.

### Prospective study

As shown in Figure 2, 58 patients were included in the prospective study. Examinations performed prior to PET/CT in these patients are reported in Table 2. A combined abdominal/thoracic CT was performed in 5 patients, whereas a thoracic CT and an abdominal CT were each performed in 1 patient. No diagnostic clues were detected on any of the CT scans.

In total, 66% of PET/CT-scans revealed non-physiological 18F-FDG uptake. Two patients showed two areas of pathological uptake that were possibly related to the elevated ESR (i.e. arthritis and non-specific colorectal uptake in one, and sarcoidosis and diverticulitis in the other).

**Figure 3:**
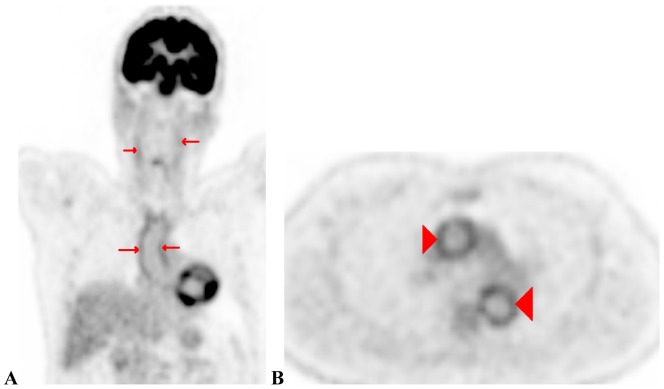
Coronal PET/CT slice showing physiological 18-FDG uptake in the brain and urogenital tract and increased 18-FDG uptake in the ascending aorta and carotid arteries (A, red arrows). Transverse PET/CT slice showing increased 18-FDG uptake in the ascending and descending aorta (B, red arrowheads).

**Figure 4:**
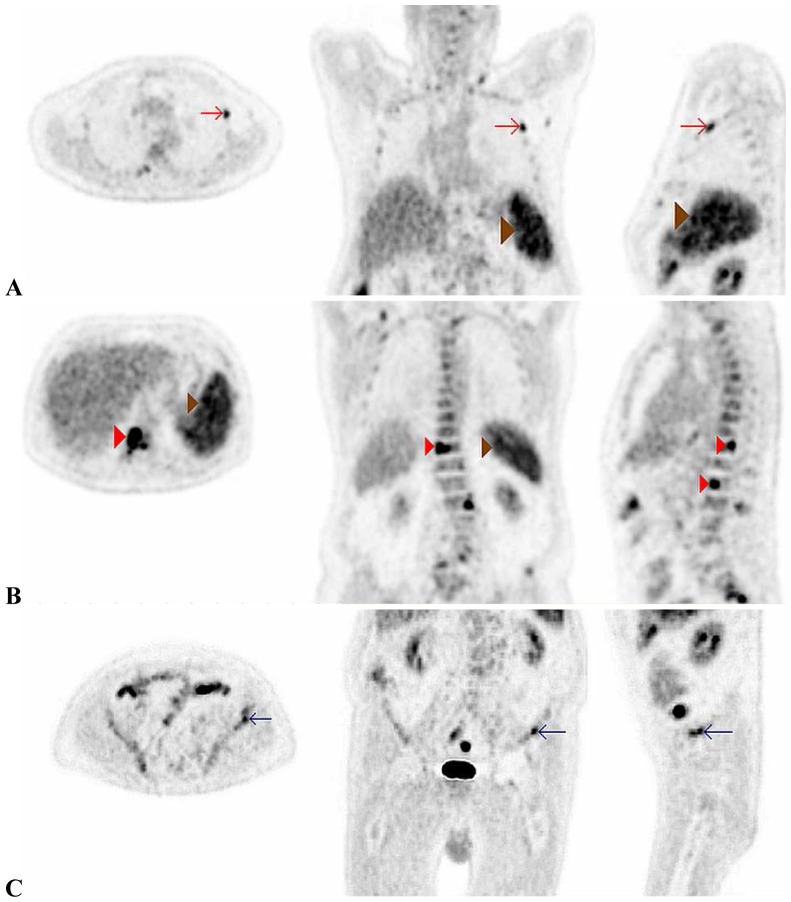
Axial, coronal and sagittal PET/CT images showing increased FDG-uptake in the rib (A, red arrow), spine (B, red arrowhead), spleen (B, brown arrowhead) and pelvic bone (C, blue arrow) suggesting lymphoproliferative disease. This PET/CT diagnosis was histologically confirmed after a bone marrow biopsy was performed.

**Figure 5:**
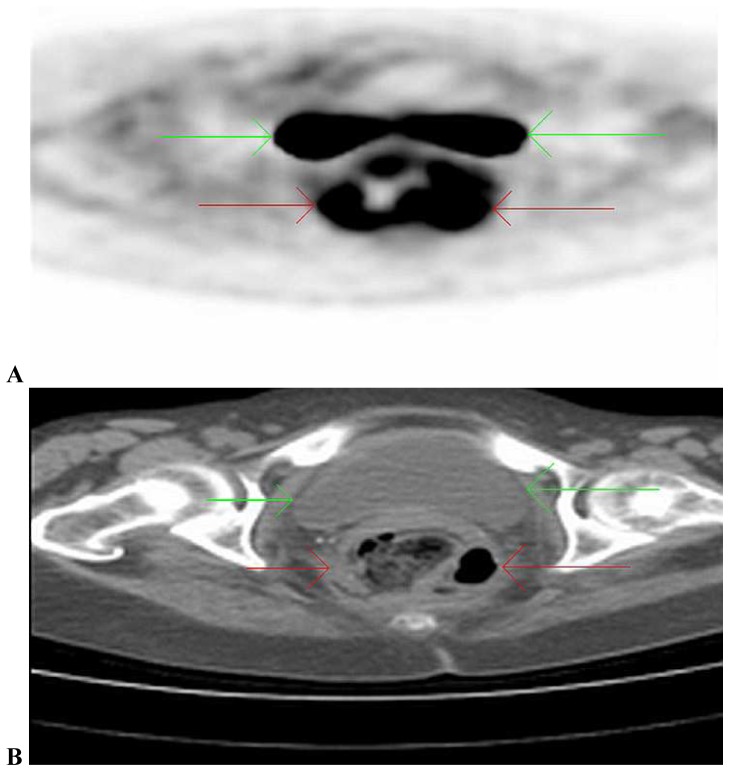
Axial PET/CT images showing physiological 18-FDG uptake in the bladder (green arrow) and increased peri-rectal 18-FDG uptake (A, red arrow). Additionally, the presence of air is detected on the low-dose CT (B). This was not present on an abdominal CT-scan that was performed prior to the PET/CT-scan. A diagnosis of peri-rectal abscess was confirmed during explorative surgery.

Non-infectious inflammation represented the largest group of abnormal scans (42% of all scans). Within this group, large-vessel vasculitis was the most frequent diagnosis (24% of all included patients). Figure 3 shows an example of a PET/CT suggesting large-vessel vasculitis. Malignancy and infection were suspected in 3 of 58 (5%) and 5 of 58 (8%) patients, respectively. Scan-results of a patient with lymforeticular malignancy and peri-rectal abscess are displayed in Figure 4 and 5. Non-specific, abnormal patterns of 18F-FDG uptake were encountered in 7 of 58 scans (12%). In only one of these 7 a diagnosis compatible with the pattern of abnormal 18-FDG uptake was obtained during follow-up.

Among the group of patients with sufficient clinical follow up data 67% of abnormal scan results were confirmed. With respect to the large-vessel vasculitis diagnosis: a temporal artery biopsy was performed in 10 of 14 patients, despite the fact that no clinical clues for temporal arteritis were present. Of the 10 biopsies, 5 turned out to be compatible with giant cell arteritis; a positive biopsy rate comparable to previous studies in large-vessel vasculitis patients.[13]–[15] As temporal artery biopsy is less sensitive in patients with large-vessel vasculitis as opposed to patients with ‘cranial’ temporal arteritis the diagnosis was confirmed in the 5 patients with a negative biopsy result by clinical follow up (i.e. prompt response to immunosuppressive therapy and no alternative diagnosis obtained).

Ten patients were lost to follow-up. Five of them (50%) had a normal PET/CT-scan and were not followed up by their treating physician. Only 1 diagnosis (‘atypical Polymyalgia rheumatica’) was established in the group of 15 patients with a normal PET/CT result and sufficient follow-up. An alternative diagnosis was established in 3 of 40 abnormal scan-results. These diagnoses were: myelodysplastic syndrome in 1 patient (scan result: non-physiological colorectal 18F-FDG uptake) and PMR in 2 patients (scan results: non-physiological urogenital 18F-FDG uptake in one and arthritis of the hips and shoulders in the other)

## Discussion

This study demonstrates that PET/CT provides potentially diagnostic clues in a substantial proportion of patients (66% in prospective study, 71% in retrospective study) with an elevated erythrocyte sedimentation rate (ESR) in whom the initial routine evaluation did not reveal a diagnosis. Moreover, the majority (67–84%) of these diagnostic clues was confirmed during follow-up. In patients with a normal PET/CT, a cause of the elevated ESR was rarely found.

Our study is the first to systematically assess the diagnostic potential of PET/CT in patients with chronic signs of inflammation and absence of clinical diagnostic clues. Currently, there are no guidelines for the evaluation of patients with elevated inflammatory markers. It seems common practice, however, to exclude common causes of an elevated ESR (e.g. infection and cancer) by performing a chest X-ray and abdominal ultrasound and by protein electrophoresis. These examinations were indeed performed in the majority of patients in our retrospective, observational study, and were performed by protocol in the prospective study. Furthermore, the ESR in our patients had to be elevated for a period of 4 weeks to exclude intercurrent conditions (e.g. viral infections). The protocol was thus designed to include particularly patients with elevated ESR *of unknown origin*.

Previous studies have systematically evaluated the diagnostic yield of 18-FDG PET in fever of unknown origin (FUO), a comparable (nevertheless, distinct) clinical problem. A MEDLINE-search, using the terms (fever of unknown origin OR FUO) AND (PET OR positron emission tomography) revealed that 160 studies have reported on the yield of 18-FDG PET, either PET-alone or combined with low-dose CT, in FUO. Overall, the diagnoses most often established by 18-FDG PET in FUO are infectious diseases (19-46%). Auto-inflammatory diseases (15–33%) and malignancies (2–25%) are encountered less frequently.[5], [7], [16]–[18]

In contrast to FUO, there are only a few reports on the diagnostic yield in patients with an elevated ESR, or other signs of inflammation, of unknown origin. Moreover, reports on the diagnostic yield of PET/CT in these patients are minimal.[1], [10], [19] One of these studies retrospectively investigated the diagnostic yield in patients with an elevated ESR and anemia.[1] Due to the retrospective nature of this study there was no systematic evaluation of these patients. Furthermore, PET/CT was not performed in these patients. A specific diagnosis was obtained in 80 of 139 patients (58%) in this study. Malignancy accounted for 22% of all diagnoses, infectious diseases for 10%, whereas collagen vascular diseases accounted for only 9% and non-neoplastic hematologic diseases for 5%.

Another study assessed the value of PET/CT in 4 patients with a prolonged inflammatory syndrome.[19] However, C-reactive protein (CRP) was used in this study to assess inflammation. PET/CT contributed to establishing a diagnosis in 2 of 4 (50%) patients, large-vessel vasculitis and gastric adenocarcinoma were diagnosed. The final study, in which the diagnostic yield between patients with FUO and inflammation of unknown origin (IUO) was compared, included 57 patients with IUO.[10] ESR was determined in 46 of these patients, whereas CRP was measured in all patients and was considered to be the primary marker of inflammation. As opposed to our study, FDG-PET(/CT) was not performed in all patients included (80%). Furthermore, 51% of all patients with IUO already used corticosteroids at the time of FDG-PET(/CT). FDG PET was helpful in establishing a diagnosis in 36% of patients with IUO. As in our study, auto-inflammatory disease was encountered most frequently. No firm diagnosis was obtained in 22 patients (39%) as compared to 24 patients (42%) in a matched group with FUO. The time to diagnosis was longer in the group of patients with IUO (mean of 29 versus 13 days).

Our results confirm the detection of cancer and infection by PET/CT in a number of cases. However, as opposed to the earlier studies in FUO, and in line with the few preliminary studies in chronic inflammation, we found a higher percentage of PET/CT results suggesting auto-inflammatory disease. In particular, a large proportion was due to large-vessel vasculitis.

Large-vessel vasculitis in elderly subjects frequently accompanies the clinical syndrome of giant cell arteritis (GCA).[20] However, isolated large-vessel vasculitis is increasingly recognised as a specific phenotype of giant cell arteritis. As opposed to temporal arteritis, signs and symptoms are often non-specific in large-vessel vasculitis and therefore, many patients present with constitutional symptoms such as weight loss, fatigue, anorexia, night sweats or FUO, but symptoms are often subtle or even absent.[14] The non-specific presentation of this type of large-vessel vasculitis, which is referred to as ‘occult’ or ‘silent’ giant cell arteritis, may cause diagnostic delay associated with multiple, sometimes invasive, diagnostic tests.[13], [21] Furthermore, aortic pathology and large-artery obstruction is not rare in these patients.[14], [22] PET/CT has contributed to the increased awareness of large-vessel vasculitis, since the aorta and its proximal branches are often affected without (symptomatic) involvement of the temporal arteries, rendering the diagnosis dependent on imaging inflammation in large arteries that are inaccessible to biopsy.

Sensitivity of temporal artery biopsy, which is far from optimal for temporal arteritis itself, is even lower in large-vessel vasculitis.[13], [14] Temporal arteries are infrequently involved and skip lesions decrease sensitivity. Additionally, large arteries are normally inaccessible for biopsy unless a vascular calamity requires surgery. Despite the risk of false-negative result, some recommend temporal artery biopsy in patients with suspected LV-GCA, as a positive result is highly specific. A negative biopsy should, however, never lead to abandonment of suspicion. Hence, reliance on typical images obtained during PET/CT or, as has been suggested, CT angiography or magnetic resonance imaging, is central to the diagnosis of large-vessel vasculitis.[23]–[26] Further diagnostic support may be a favourable response to corticosteroids, however non-specific this may be.

The exact prevalence/incidence of ‘occult’ GCA is unknown. Our study suggests that it may be significantly higher than previously thought. This is in line with several post-mortem series suggesting that large-vessel GCA is much more prevalent than would be expected based on the incidence of classic temporal arteritis.[27]–[29]

Compared to FUO, the rate of infections detected by PET/CT in both our studies (8%-10%) is low. Apparently, IUO is a rarer manifestation of infectious disease than is FUO.

The results concerning the yield of malignant disease (5-27%) from the present study are comparable to previously reported FUO studies (2–25%).[5], [7], [16]–[18] The difference between the retrospective and the prospective group in the yield of malignant diseases was unexpected. This difference was not explained by a difference in diagnostic procedures performed prior to the PET/CT scan, and could be the result of chance alone.

It should be stressed that, even if PET/CT is positive, it’s result is not necessarily decisive. False-positive diagnoses occurred in 7,5 to 10% of patients with an elevated ESR and illustrate that a positive PET/CT result remains to be interpreted in the context of clinical presentation and results of additional testing. Also, whether the identification of diseases by PET/CT improves clinical outcome, or just creates lead-time bias, is not known. Therefore, future studies should focus on the outcome of these patients, especially whether an early diagnosis prevents future vascular events (e.g. aortic aneurysm rupture or dissection) in patients with large-vessel vasculitis and whether mortality is decreased in patients in whom a malignancy is detected.

Approximately 30% of PET/CT-scans performed were considered to have a normal 18F-FDG distribution pattern. This proportion might have been lower if we would have used more strict inclusion criteria, e.g. a higher cut-off value of ESR. A post-hoc analysis revealed that the ESR was significantly higher in the group with abnormal PET/CT-scans (90 (±23) vs 78 (±21) mm/h, p = 0,01). Therefore, a higher ESR cut-off value probably would probably result in a lower rate of negative PET/CT-scans. Conversely, however, diagnoses will be missed if PET/CT is only used for patients with an exceptionally high ESR. Furthermore, a normal PET/CT-scan in these patients appears somewhat reassuring, as a specific diagnosis was established in only a small percentage of patients during follow-up. These results are comparable to those found in FUO patients with a normal PET/CT-scan.[5], [30] It has to be stressed, though, that several diseases in which an elevated ESR is present may go undetected by PET/CT imaging, particularly (chronic) renal disease.[31] Therefore, renal dysfunction should be excluded as a cause of an elevated ESR, preferably prior to PET/CT imaging.

Our study has strengths and limitations. First, we systematically evaluated the diagnostic yield of PET/CT in consecutively eligible patients. In addition, the multi-center approach involving community hospitals may have limited bias associated with tertiary referral. The similarity between results of the retrospective and prospective approach provides further support for generalizability of the conclusions.

A limitation of this study is that selection started only after clinicians concluded that routine work-up was non-diagnostic, and reported this to the study coordinators. Had we registered all patients being referred to participating centers for elevated ESR, we would have been able to better describe the fraction, selection, and distinguishing characteristics of patients we selected for PET/CT among all referred patients. Even then, however, we would miss the data from general practitioners, where obviously selection takes place in terms of referral to the hospital. Future studies should attempt to grasp the entire process from general practitioners' offices to the qualification ' unknown origin' by hospital specialists. Another consideration is the small proportion of patients in whom morphological imaging (i.e. by conventional CT scan ) has been performed prior to the PET/CT scan. This approach may have precluded the use of PET/CT in several cases if morphological abnormalities were detected that may have caused the elevated inflammatory parameters. Moreover, investigators studying FUO have stated that an abdominal CT scan should be one of the first investigations as it detects intra-abdominal abscesses and lymphoproliferative disease.[32] The wide (and quick) availability and relatively low cost further support this approach. Furthermore, abnormal results of chest (not abdominal) CT were predictive of reaching a diagnosis in FUO patients.[6] MRI may also be of value but this is less well studied in FUO.[33]

Nevertheless, choosing the optimal diagnostic strategy (including when and how to image) is hampered by the lack of controlled trials regarding the diagnostic yield of differing imaging modalities in patients with fever or inflammation of unknown origin.[6] Several strengths of PET/CT imaging, compared to conventional CT, are: 1) less radiation exposure, 2) higher sensitivity and specificity,[6], [34] (however, the retrospective nature of one of these studies impedes generalizability), 3) better discrimination of active infectious or inflammatory lesions from residual anatomical changes due to cured processes or surgery (Figure 4) and 4) acquisition of whole-body imaging, whereas CT and MRI routinely provide only information on a part of the body.[35]

Finally, both our own clinical experience and results from FUO studies show that temporal (giant cell) arteritis accounts for a substantial proportion of causes of fever or inflammation of unknown origin in elderly patients.[32]

Another limitation is the absence of formal criteria for visual assessment of PET/CT-scan images. Also, there are no consensus criteria or formal guidelines for the diagnosis of large-artery giant cell arteritis. The American College of Rheumatology classification criteria for giant cell arteritis are essentially aimed at classifying temporal arteritis, not large-vessel vasculitis.[36] Also, formal criteria for interpreting and classifying enhanced 18F-FDG uptake in large arteries are lacking. In clinical practice, visual assessment of the intensity and distribution of 18F-FDG uptake is important for distinguishing vasculitis from severe atherosclerosis, which may also enhance vascular 18F-FDG uptake.[37] On the other hand, clinical follow up (i.e. fast improvement of symptoms and laboratory features in patients treated with steroids) supported the diagnosis in all cases diagnosed with large-vessel vasculitis. Experience has been considered to be an important contributor to the correct diagnosis of large-vessel vasculitis on PET/CT.[38]

In our studies, PET/CT-scans were performed as part of clinical care and were therefore assessed by local nuclear medicine physicians. Observers were not blinded for clinical data and scans were not assessed by an independent second observer. This may be considered a limitation. On the other hand, it enhances the external validity of our findings, since central and blinded scan readings are not part of clinical practice. Also, central readings by independent observer(s) are risky in the context of absence of formal diagnostic criteria, increasing the chance of biased scan interpretations. Taken together, we considered it most appropriate to perform the studies the way we did, and make essential PET/CT images available to readers.

In conclusion, PET/CT appears to be a valuable tool in the diagnostic work-up of elderly patients with an elevated ESR of unknown origin in whom the initial routine evaluation does not provide potentially diagnostic clues. Images compatible with large-vessel vasculitis may be a particularly prevalent result of PET/CT. Independent studies are required to confirm our findings, to define the optimum diagnostic stage in which PET/CT is employed (e.g. is morphological imaging including chest and abdominal CT or MRI indicated prior to a PET/CT scan), and to address the cost-effectiveness of different diagnostic strategies (including early- or late-stage PET/CT) is this clinical context.
